# Integrated Analysis of Metabolites and Biological Endpoints Bring New Insights into Sulfamethoxazole Stress Tolerance in Ryegrass

**DOI:** 10.3390/plants14040538

**Published:** 2025-02-10

**Authors:** Yong Yang, Jiangtao Jia, Tao Han, Heng Zhang, Yvjie Wang, Luying Shao, Xinyi Wang

**Affiliations:** 1 Henan Joint International Laboratory for Crop Multi-Omics Research, School of Life Sciences, Henan University, No. 85 Jinming Road, Kaifeng 475004, China; 104752160097@vip.henu.edu.cn (Y.Y.); jjt0821@126.com (J.J.); xhzhang17@163.com (H.Z.); yujie.wang@henu.edu.cn (Y.W.); shaoluying@henu.edu.cn (L.S.); wangxinyi000831@163.com (X.W.); 2 National Key Laboratory of Cotton Bio-Breeding and Integrated Utilization, Henan University, No. 85 Jinming Road, Kaifeng 475004, China; 3 School of Horticulture and Landscape Architecture, Henan Institute of Science and Technology, Xinxiang 453003, China

**Keywords:** biological endpoints, metabolites, ryegrass, sulfamethoxazole

## Abstract

Although metabolomics is widely used to assess the detrimental effects of antibiotics and characterize stress response, the relationships between metabolites and biological endpoints following antibiotics remain unknown. In our study, we exposed ryegrass seeds to sulfamethoxazole for five days. The results showed that sulfamethoxazole inhibited plant growth (by 12.90–85.83%). It also decreased chlorophyll content (by 35.40–93.32%), carotenoid content (by 32.76–90.18%), and root cell permeability (by 98.43–99.29%), but increased root reactive oxygen species (ROS) concentration (increasing rate: 11.32- to 137.36-times). Moreover, high sulfamethoxazole concentrations increased superoxide dismutase (SOD), peroxidase (POD), and catalase (CAT) activities. To elucidate the association between metabolites and biological endpoints, we conducted an orthogonal partial least squares analysis. The results showed that sulfamethoxazole significantly altered six metabolic pathways. Among the metabolites modulated by sulfamethoxazole, amino acids mainly affected root growth and ROS concentration, whereas carbohydrates were substantially associated with the effect of sulfamethoxazole on cell permeability. Many metabolites had contrasting effects. For example, some metabolites increased root fresh weight and improved cell permeability by decreasing ROS levels and SOD, POD, and CAT activities. By contrast, some metabolites negatively affected root fresh weight and cell permeability by increasing ROS levels and SOD, POD, and CAT activities. These observations bring new insights into ryegrass responses to sulfamethoxazole-induced stress.

## 1. Introduction

Microorganisms, animals, and plants produce metabolites called antibiotics during their life cycles. These metabolites have disease resistance-related or other bioactive properties that can hinder the growth of other living cells. In terms of human and animal medicine, antibiotics are the third most administered drug, accounting for more than 70% of the veterinary drug market [1]. In the field of animal husbandry and breeding, veterinary drugs, such as antibiotics and antiparasitic compounds, are frequently used to prevent and treat various diseases [2,3]. However, the over-reliance on concentrated feeding patterns and the failure to stop administering antibiotics to livestock within the prescribed period before sale can lead to excessive levels of antibiotics in livestock, with potentially adverse effects on food safety [4]. Although major countries and regions, including the European Union, the USA, and China, have banned the use of antibiotics in animal feed, in practice, antibiotic residues remain detectable [5]. In addition, with the production and consumption of antibiotics increasing annually, their effects on the environment are becoming increasingly serious. Antibiotics pose a threat to human health [6], but they may also disrupt normal plant and animal development and metabolism [7]. For example, in plant ecosystems, antibiotics can significantly inhibit the growth of plant roots, implying they may serve as an important biological indicator of plant toxicity [8].

As one of the key elements of systems biology, metabolomics has gradually emerged, followed closely by the development of genomics and proteomics [9]. Within each living cell, there are many active life cycle-related processes, most of which occur at the level of metabolites. For example, signal transmission, energy flow, and communication between cells are all carefully controlled by metabolites. Cellular environmental characteristics may be thoroughly understood by analyzing changes in metabolites, which may be influenced by several factors, including the nutritional status of the cell, drugs, and environmental pollutants. Notably, certain metabolites can act as biomarkers of responses to environmental stimuli [10], revealing how these stimuli affect biological activity [11]. Therefore, by studying metabolites, we can gain insights into active biological events. However, in terms of antibiotic toxicity, the relationships between metabolites and biological endpoints remain relatively undetermined.

Jin [12] reported that low sulfamethoxazole concentrations can promote the growth of Chinese cabbage and rice to some extent, but high sulfamethoxazole concentrations have an inhibitory effect on seedling growth. Another study showed that sulfamethoxazole increases leaf length in spinach but has no significant effect on basil and coriander leaf length [13]. In rapeseed seedlings, sulfamethoxazole promotes the production of many reactive oxygen species (ROS) [14] in a concentration-dependent manner. Moreover, sulfamethoxazole can increase phenolic acid, flavonoid, carbohydrate, and amino acid metabolite contents, resulting in changes in phenylpropanoid [14], carbohydrate, and amino acid metabolic pathways [12]. However, there are no systematic studies that explored the effects of sulfamethoxazole on plant metabolites and biological endpoints. The objectives of this study were as follows: (1) to assess the extent of the effects of sulfamethoxazole on ryegrass growth and development; (2) to combine the results of a metabolic analysis and biological endpoints to further explore the mechanism underlying sulfamethoxazole toxicity; and (3) to detect changes in metabolites following exposure to sulfamethoxazole and reveal the metabolic pathways linking these changes to biological endpoints.

## 2. Results and Discussion

### 2.1. Morphological Characteristics

**Figure 1:**
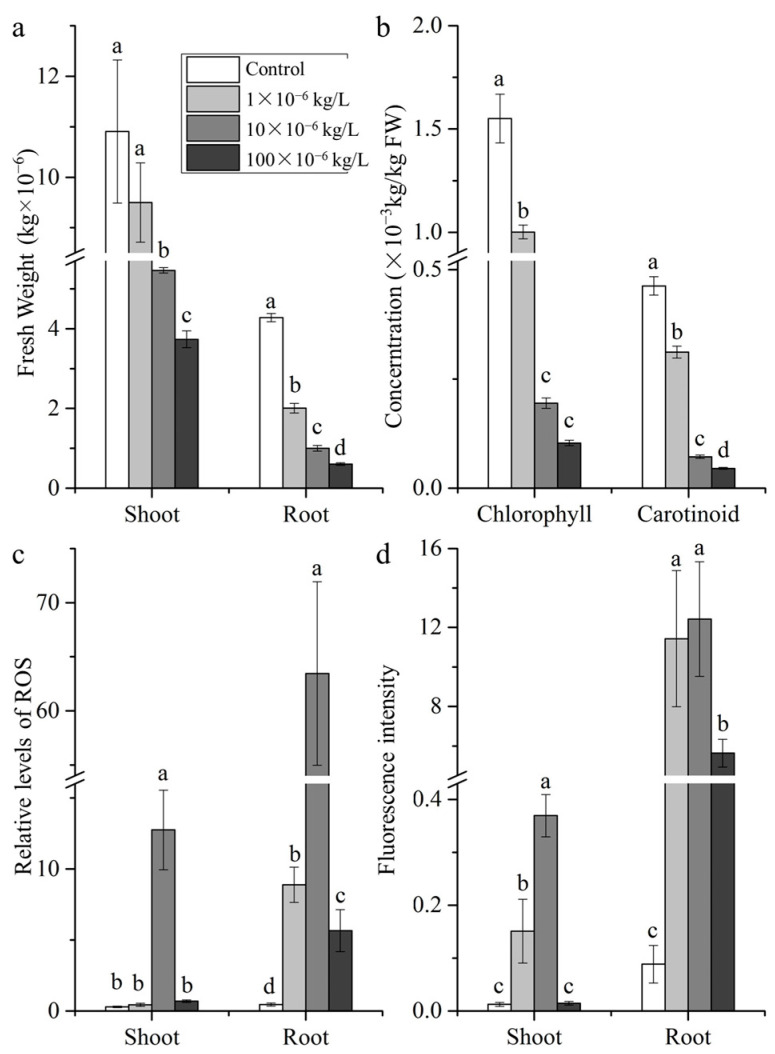
Morphological characters and oxidant stress: (**a**) fresh weight; (**b**) chlorophyll and carotenoid; (**c**) ROS levels; (**d**) cell permeability. Different letters indicate significant differences at *p* < 0.05. n = 3.

The effects of sulfamethoxazole on ryegrass were studied in detail (Figure 1). Sulfamethoxazole inhibited ryegrass growth, with the inhibitory effects increasing as the sulfamethoxazole concentration increased (Figure 1a and Figure S1). The shoot fresh weight was significantly lower after the 10 × 10^−6^ and 100 × 10^−6^ kg/L sulfamethoxazole treatments than after the control treatment. Root fresh weights following the 1 × 10^−6^, 10 × 10^−6^, and 100 × 10^−6^ kg/L sulfamethoxazole treatments were significantly lower than that following the control treatment. Sulfamethoxazole has a strong inhibitory effect on microalgae proliferation and growth of *Chlorella vulgaris* [15,16]. The 10 × 10^−6^ kg/L sulfamethoxazole treatment decreased the shoot weight by 49.88% (relative to the control shoot weight), but the decrease in shoot weight was even greater (65.77%) following the 100 × 10^−6^ kg/L sulfamethoxazole treatment. The 1 × 10^−6^, 10 × 10^−6^, and 100 × 10^−6^ kg/L sulfamethoxazole treatments also decreased the root weight by 53.12%, 76.64%, and 85.83%, respectively (relative to the control root weight).

When seedlings were exposed to sulfamethoxazole, the shoot chlorophyll content decreased significantly by 35.40–93.32% (Figure 1b), with greater decreases at higher sulfamethoxazole concentrations. This is in accordance with the findings of an earlier study that showed sulfamethoxazole decreases chlorophyll contents in *P. tricornutum* and *Chlorella vulgaris* [15,16]. Chlorophyll contents also decrease in wheat following treatments with increasing sulfamethoxazole concentrations [17]. Additionally, a comparison with the control group revealed the carotenoid content decreased by 32.76–90.18% in sulfamethoxazole-treated seedlings, with greater decreases at higher sulfamethoxazole concentrations.

### 2.2. ROS and Cell Permeability

Sulfamethoxazole treatments significantly increased ROS production in plant roots (Figure 1c). Specifically, ROS levels were 19.37-, 138.26-, and 12.32-times higher in the 1 × 10^−6^, 10 × 10^−6^, and 100 × 10^−6^ kg/L sulfamethoxazole-treated samples than in the controls, respectively. Similarly, 10 × 10^−6^ kg/L sulfamethoxazole treatment substantially increased shoot ROS production. The relative ROS levels after the 1 × 10^−6^, 10 × 10^−6^, and 100 × 10^−6^ kg/L sulfamethoxazole treatments were 1.46-, 42.27-, and 2.31-times higher than the control level, respectively. According to earlier research, chloroplasts are the main producers of reactive oxygen species, which is mainly because of the extremely high oxygen concentration in green leaf during photosynthesis [7,18]. Our experimental results showed that the chlorophyll content was significantly lower in sulfamethoxazole-treated plants than in control plants. The application of sulfamethoxazole stimulated the ROS synthesis in plant roots, implying ROS can be produced via non-chloroplast pathways. Previous studies found that ROS concentration under environmental stress can be increased through the mitochondria–ROS channel [7,19]. Serrander et al. [20] found that antibiotics can rapidly increase the amount of NOX4 mRNA, thereby driving the production of ROS.

In this study, we analyzed the effect of sulfamethoxazole on cell permeability (Figure 1d). In terms of plant roots, fluorescence intensities were significantly greater in the sulfamethoxazole-treated samples than in the control. Specifically, fluorescence intensities were 128.94-, 140.14-, and 63.59-times higher for the samples treated with 1 × 10^−6^, 10 × 10^−6^, and 100 × 10^−6^ kg/L sulfamethoxazole than for the control, respectively. Analyses of plant shoots revealed that fluorescence intensities were significantly higher after the 1 × 10^−6^ and 10 × 10^−6^ kg/L sulfamethoxazole treatments than after the control treatment (11.92- and 29.16-times higher, respectively). By contrast, the fluorescence intensity following the 100 × 10^−6^ kg/L sulfamethoxazole treatment was only 1.16-times higher than that following the control treatment.

### 2.3. SOD, POD, and CAT Activities

**Figure 2:**
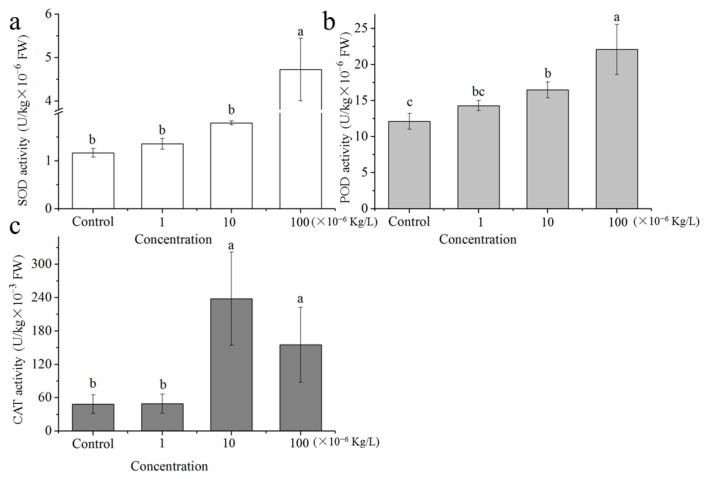
Antioxidant activities: (**a**) SOD activity; (**b**) POD activity; (**c**) CAT activity. Different letters indicate significant differences at *p* < 0.05. n = 3.

Superoxide dismutase (SOD) plays a key role in regulating the redox balance in plant cells. In this study, 1 × 10^−6^ and 10 × 10^−6^ kg/L sulfamethoxazole treatments had limited effects on SOD activity (Figure 2a), whereas the 100 × 10^−6^ kg/L sulfamethoxazole treatment resulted in a significant increase in SOD activity. More specifically, SOD activity was 1.16-, 1.53-, and 4.04-times higher in samples treated with 1 × 10^−6^, 10 × 10^−6^, and 100 × 10^−6^ kg/L sulfamethoxazole than in the control, respectively. Previous research also indicated sulfamethoxazole can promote SOD activity [15,16,17]. Thus, in response to sulfamethoxazole stress, protective mechanisms may be triggered in ryegrass to decrease the damages due to free radicals.

Increases in the sulfamethoxazole concentration were accompanied by increases in POD activity (Figure 2b). Although 1 × 10^−6^ kg/L sulfamethoxazole had no significant effect on POD activity, 10 × 10^−6^ and 100×10^−6^ kg/L sulfamethoxazole significantly enhanced POD activity. Specifically, POD activities were 1.18-, 1.36-, and 1.82-times higher following the 1 × 10^−6^, 10 × 10^−6^, and 100 × 10^−6^ kg/L sulfamethoxazole treatments than following the control treatment, respectively.

In plants, excessive oxidative stress leads to the increased production of ROS, which are scavenged via activated antioxidant systems. This scavenging is associated with enhanced SOD and POD activities [21,22]. In the present study, both SOD and POD activities were higher after the 100 × 10^−6^ kg/L sulfamethoxazole treatment than after the 10 × 10^−6^ kg/L sulfamethoxazole treatment. Thus, the scavenging of ROS by these antioxidative enzymes may increase under high environmental stress conditions.

There were no significant differences in the catalase (CAT) activities of the control and 1 × 10^−6^ kg/L sulfamethoxazole-treated samples (Figure 2c). However, treatments with 10 × 10^−6^ and 100 × 10^−6^ kg/L sulfamethoxazole significantly increased CAT activity, which is consistent with the findings of an earlier study on the effect of sulfamethoxazole on CAT activity [15].

### 2.4. Metabolite Analysis

**Figure 3:**
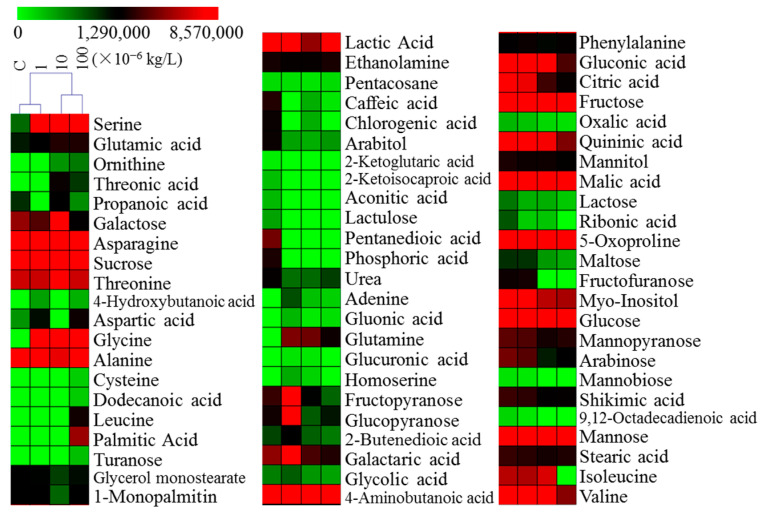
Metabolite changes. Heatmap showing the relative contents of metabolites.

After an in-depth analysis, we determined that key indices, including plant root weight, ROS content, cell permeability, SOD, POD, and CAT activities, reflected the sensitivity to sulfamethoxazole. In addition, we examined plant metabolite dynamics using metabolomics techniques. Specifically, we comprehensively analyzed each sample using gas chromatography and mass spectrometry systems [7], which identified 68 metabolites, including amino acids, carbohydrates, lipids, organic acids, and other compounds (Figure 3). On the basis of a hierarchical cluster analysis, we divided the sulfamethoxazole treatment groups into two main categories: C/1 × 10^−6^ kg/L and 10 × 10^−6^/100 × 10^−6^ kg/L. The results revealed that high sulfamethoxazole concentrations may significantly alter metabolic activities in ryegrass cells. Common and unique metabolites are important markers under sulfamethoxazole stress [23]. Therefore, we generated a Venn diagram to identify common and unique metabolites among the C, 1 × 10^−6^, 10 × 10^−6^, and 100 × 10^−6^ kg/L treatment groups (Figure S2). There were 39 metabolites that were common to all four groups, whereas six and five metabolites were unique to the C and 100 × 10^−6^ kg/L groups, respectively.

**Figure 4:**
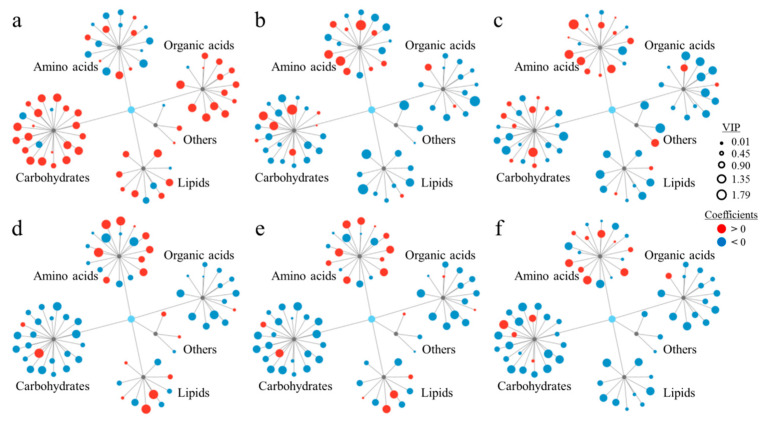
Connections between metabolite changes and biological endpoints induced by sulfamethoxazole: (**a**) root fresh weight; (**b**) ROS levels; (**c**) cell permeability; (**d**) SOD activity; (**e**) POD activity; (**f**) CAT activity. Red and blue represent positive and negative coefficients, respectively. The sizes of circles represent VIP values.

We used an orthogonal partial least squares method to evaluate the potential relationship between plant root metabolites and biological endpoints (Figure 4). The relative contribution of metabolites to biological endpoints was assessed by calculating VIP (Variable Importance Projection) values. For root fresh weight (Figure 4a), 34 metabolites had a VIP value greater than 1, indicating that they significantly influenced the fresh weight of plant roots. We further divided these metabolites according to how they affect the root fresh weight. Specifically, 49 metabolites had positive effects (coefficient > 0) on root fresh weight, whereas 19 metabolites had negative effects (coefficient < 0). Thirteen metabolites, including fructose, citric acid, maltose, mannose, and so on, had significantly positive effects on root fresh weight (VIP value > 1, coefficient > 0) (Figure 4a). By contrast, 32 metabolites, such as ornithine, glutamate, glycine, serine, and so on, had significantly negative effects (VIP value < 1, coefficient < 0).

The relationships between changes in root metabolites and other biological endpoints were analyzed further (Figure 4b–f). We found that 28, 29, 30, 32, and 31 metabolites positively affected ROS levels, cell permeability, SOD activity, POD activity, and CAT activity, respectively. Some of the remaining metabolites had negative effects on ROS levels, fluorescence intensity, SOD activity, POD activity, and CAT activity. Ten metabolites, including threonine, sucrose, asparagine, serine, and so on, had significantly positive effects on the ROS concentration (VIP value > 1, coefficient > 0). However, 18 metabolites, including stearic acid, mannopyranose, phenylalanine, glucose, and so on, had significantly negative effects on the ROS concentration (VIP value < 1, coefficient < 0) (Figure 4b). In addition, cell permeability is reportedly negatively correlated with fluorescence intensity [24]. In total, 8 metabolites, including glutamine, glucuronic acid, serine, asparagine, and so on, had significantly negative effects on cell permeability (VIP value > 1, coefficient > 0), but 21 metabolites, including urea, arabitol, stearic acid, mannose, and so on, had significantly positive effects on cell permeability (VIP value < 1, coefficient < 0) (Figure 4c). In total, 10 metabolites, including cysteine, dodecanoic acid, leucine, palmitic acid, and so on, had significantly positive effects on SOD activity (VIP value > 1, coefficient > 0), while 20 metabolites, including quininic acid, gluconic acid, mannitol, fructose, and so on, had significantly negative effects on SOD activity (VIP value < 1, coefficient < 0) (Figure 4d). In total, 9 metabolites, including cysteine, dodecanoic acid, leucine, palmitic acid, and so on, had significantly positive effects on POD activity (VIP value > 1, coefficient > 0), whereas 23 metabolites, including quininic acid, gluconic acid, mannitol, fructose, and so on, had significantly negative effects on POD activity (VIP value < 1, coefficient < 0) (Figure 4e). Six metabolites, including threonic acid, glutamic acid, ornithine, serine, and so on, had significantly positive effects on CAT activity (VIP value > 1, coefficient > 0). By contrast, 25 metabolites, including mannopyranose, glucose, mannobiose, stearic acid, and so on, had significantly negative effects on CAT activity (VIP value < 1, coefficient < 0) (Figure 4f).

Notably, metabolites that negatively affected root fresh weight and cell permeability positively affected ROS levels as well as SOD, POD, and CAT activities. Moreover, metabolites that positively affected root fresh weight and cell permeability negatively affected ROS levels as well as SOD, POD, and CAT activities. Thus, we established a new association model that has further elucidated the mechanism underlying specific biological endpoints, while also being useful for verifying the effects of environmental stress.

**Figure 5:**
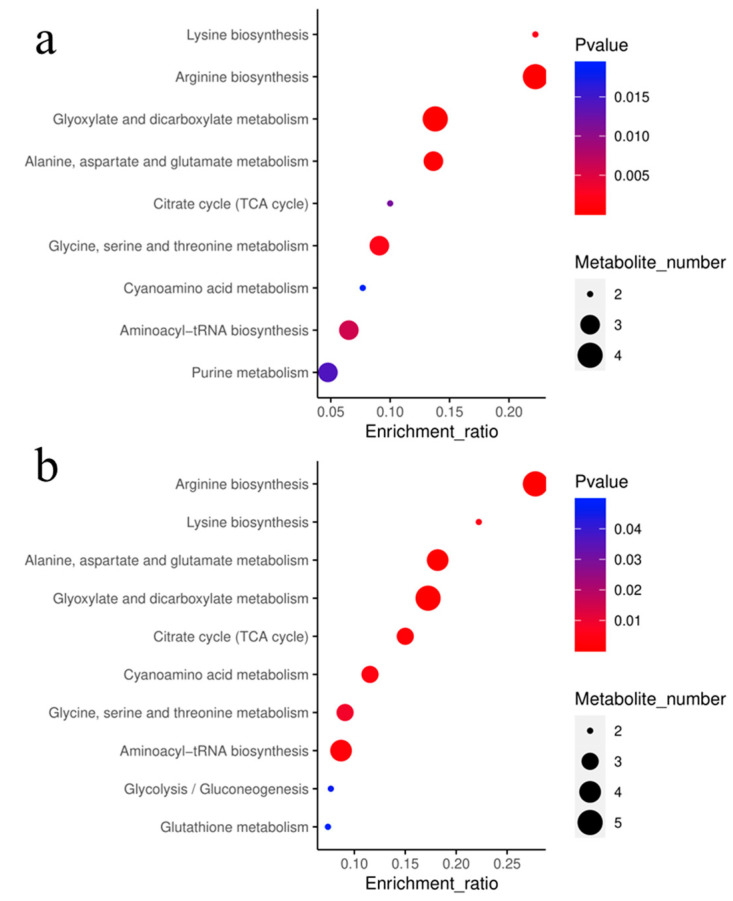

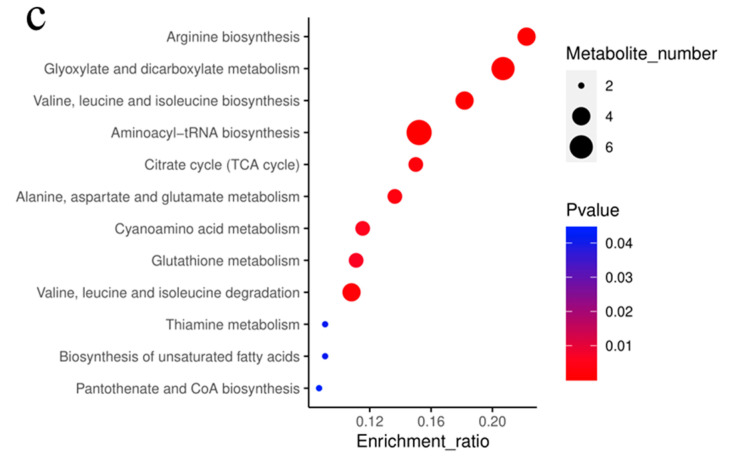
Kyoto encyclopedia of genes and genomes (KEGG) enrichment pathways of differential metabolites: (**a**) C vs. 1 × 10^−6^ kg/L; (**b**) C vs. 10 × 10^−6^ kg/L; (**c**) C vs. 100 × 10^−6^ kg/L.

**Figure 6:**
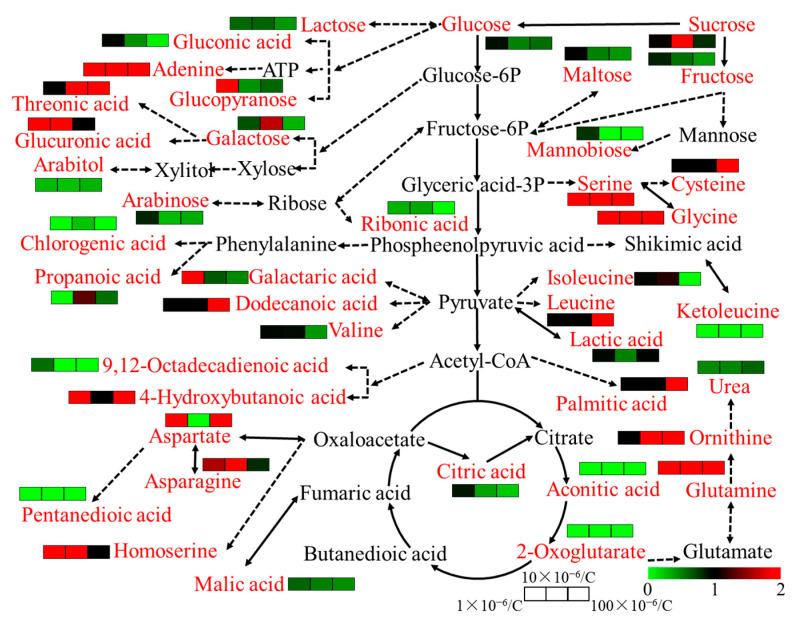
Metabolic map. Red words represent differential metabolites. The color rectangles represent up-regulation and down-regulation of metabolites content in 1 × 10^−6^, 10 × 10^−6^, and 100 × 10^−6^ kg/L sulfamethoxazole treatments compared with the control, respectively.

Furthermore, differentially abundant metabolites were identified, using a 2-fold difference between the control and treated samples as the threshold. According to MetaboAnalyst, sulfamethoxazole affected the following pathways in the root: arginine biosynthesis; glyoxylate and dicarboxylate metabolism; alanine, aspartate, and glutamate metabolism; citrate cycle (TCA cycle); aminoacyl-tRNA biosynthesis; and cyanoamino acid metabolism (Figure 5). By modulating these metabolic pathways, sulfamethoxazole can influence the final state of the treated plant. To better demonstrate the potential effects of sulfamethoxazole on ryegrass biological endpoints, we visualized the changes in metabolites (Figure 6). Notably, our results indicate that sulfamethoxazole can effectively inhibit root growth and stimulate ROS production, which are closely related to amino acid metabolism [24]. In addition, earlier research showed that exposure to environmental stress can lead to increases and decreases in amino acid synthesis [25,26]. Carbohydrates have important effects on cell permeability [27]. In the current study, lactose, lactulose, and mannose contents were negatively related to fluorescence intensity. Hence, sulfamethoxazole decreases cell permeability via carbohydrate degradation. We also observed that increases in glutamine levels lead to decreases in cell permeability, which is in accordance with the results of an earlier study by Hu et al. [27]. Therefore, we further clarified the mechanism underlying the toxicity of sulfamethoxazole on the basis of a combined analysis of metabolites and biological endpoints.

## 3. Materials and Methods

Ryegrass seeds were soaked in a 2% H_2_O_2_ solution for 15 min and then washed three times with ultra-pure water. The surface-sterilized seeds were placed in Petri dishes 4.5 cm diameter and treated with different sulfamethoxazole concentrations (0, 1 × 10^−6^, 10 × 10^−6^ and 100 × 10^−6^ kg/L), with 3 replicates per concentration (n = 3). Petri dishes were placed in an illumination incubator [16 h light (6000 lux; 25 °C)–8 h dark (18 °C) cycle with 70% relative humidity] for 5 days. Plant shoots were dipped in an 8.0 mL ethanol–acetone–water (45:45:10) solution. After seven days, the absorbance (470, 645, and 663 nm) of the extract was measured using a spectrophotometer. The absorbance data were used to calculate the chlorophyll and carotenoid contents as previously described [24].

### 3.1. ROS Levels and Cell Permeability

We used 2′,7′-dichlorodihydrofluorescein diacetate (DCFH-DA) to react with ROS to produce dichlorofluorescein. Notably, DCFH-DA is non-polar, hydrophobic, and non-fluorescent, enabling it to easily enter cells. Cell permeability was analyzed using fluorescein diacetate. Specifically, ryegrass shoots and roots were washed three times and then soaked in fluorescein diacetate for 40 min at 20 °C in darkness. Samples were washed three times with ultra-pure water. We used a fluorescence microscope to observe and photograph stem and root tips (200× magnification), after which Image J 1.43 software was used to measure relative fluorescence intensity [7,24].

### 3.2. SOD, POD, and CAT Activity

After five days of treatment, we obtained fresh root samples and ground them in 62.5 mM cold phosphate buffer. The ground material was centrifuged at 15,000× *g* for 10 min at 4 °C. The supernatant was collected for analyses of SOD, POD, and CAT activities as described by Han et al. [7]. The absorbances of mixture 1 (phosphate buffer, riboflavin, methionine, EDTA-Na_2_, NBT, and enzyme solution), mixture 2 (guaiacol, H_2_O_2_, and enzyme solution), and mixture 3 (phosphate buffer, deionized water, H_2_O_2_, and enzyme solution) were determined at 560, 470, and 240 nm, respectively. SOD, POD, and CAT activities were calculated according to the absorbances.

### 3.3. Metabolites

Ryegrass roots were frozen in liquid nitrogen and then ground in a 2 mL solution comprising chloroform, water, and methanol (1:1:2.5). Root cells were lysed via ultrasonication, and then centrifuged at 11,000× *g* 4 °C. These steps were repeated. The collected supernatants were combined and added to 0.8 mL water, with the resulting solution centrifuged at 5000× *g* for 3 min. Samples were dried using nitrogen gas and freeze-dried.

Dried metabolites were mixed with 50 μL methoxamine hydrochloride (20 mg/mL) and then oscillated, centrifuged, and incubated at 30 °C for 90 min. After adding 80 μL N-methyl-N-(trimethylsilyl) trifluoroacetamide, the mixture was oscillated, centrifuged, and incubated at 37 °C for 30 min. Finally, 1 μL derivatized sample was injected into a HP-5ms (30 m) gas chromatography column. Metabolites were detected using a non-fission gas chromatography–mass spectrometry system. The injection port and the delivery line temperatures were set to 230 °C and 250 °C, respectively. The temperature program was as follows: 80 °C for 2 min and then increased to 325 °C at 15 °C/min. We used the intensities in the NIST 14.0 mass spectrometry library to verify our experimental results [7].

### 3.4. Statistical Analysis

Data were analyzed using SPSS 16.0 software. To test the homogeneity of variance, we used a one-way analysis of variance along with the least significant difference test (root fresh weight, carotenoid, cell permeability, and POD and CAT activity) or Dunnett’s C analysis (leaf fresh weight, chlorophyll, ROS levels, and SOD activity) to determine the significant difference (*p* less than 0.05). Data are presented herein as the mean and its standard deviation. We also used SIMCA 14.1 software for cluster analysis and MeV 4.9.0 software to generate graphs.

## 4. Conclusions

Metabolomics-based analyses can provide a comprehensive view of the toxic effects of sulfamethoxazole and clarify potential associations between metabolites and biological endpoints. In our study, we elucidated the effects of sulfamethoxazole on ryegrass growth and metabolic processes, while also revealing how the affected metabolites are related to biological endpoints. Our findings reflected sulfamethoxazole-induced changes to metabolites. Interestingly, 9,12-octadecadienoic acid played a leading role in regulating root weight and cell permeability; however, it also had a significantly negative effect on ROS levels as well as SOD, POD, and CAT activities. Notably, some metabolites may increase the root fresh weight and positively affect cell permeability by decreasing the ROS level and SOD, POD, and CAT activities. Conversely, some metabolites may adversely affect the root fresh weight and cell permeability by increasing the ROS level and SOD, POD, and CAT activities. Overall, this study on ryegrass has deepened our understanding of the metabolic response to antibiotic-related toxicity. Furthermore, we established a link between the changes in root metabolites and biological endpoints, thereby clarifying the mechanism underlying the toxic effects of antibiotics.

## Data Availability

The main results and Supplementary Data have already been presented in the manuscript, and the original data can be obtained from the corresponding author on reasonable request.

## Supplementary Materials

The following supporting information can be downloaded at: https://www.mdpi.com/article/10.3390/plants14040538/s1, Figure S1: Seedling phenotypes. Figure S2: Venn analysis of metabolites.

## References

[1] Halling-Sørensen B., Nors Nielsen S., Lanzky P.F., Ingerslev F., Holten Lützhøft H.C., Jørgensen S.E. (1998). Occurrence, fate and effects of pharmaceutical substances in the environment—A review. Chemosphere.

[2] Hassouan M.K., Ballesteros O., Taoufiki J., Vílchez J.L., Cabrera-Aguilera M., Navalón A. (2007). Multiresidue determination of quinolone antibacterials in eggs of laying hens by liquid chromatography with fluorescence detection. J. Chromatogr. B.

[3] Goetting V., Lee K.A., Tell L.A. (2011). Pharmacokinetics of veterinary drugs in laying hens and residues in eggs: A review of the literature. J. Vet. Pharmacol. Ther..

[4] Gajda A., Posyniak A. (2015). Doxycycline depletion and residues in eggs after oral administration to laying hens. Food Addit. Contam. Part A.

[5] Marmulak T., Tell L.A., Gehring R., Baynes R.E., Vickroy T.W., Riviere J.E. (2015). Egg residue considerations during the treatment of backyard poultry. J. Am. Vet. Med. Assoc..

[6] Zakordonets L., Tolstanova G., Yankovskiy D., Dyment H., Kramarev S. (2016). Different regimes of multiprobiotic for prevention of immediate and delayed side effects of antibiotic therapy in children. Res. J. Pharm. Biol. Chem. Sci..

[7] Han T., Liang Y.P., Wu Z.N., Zhang L., Liu Z.W., Li Q.F., Chen X.J., Guo W.L., Jiang L.N., Pan F.F. (2019). Effects of tetracycline on growth, oxidative stress response, and metabolite pattern of ryegrass. J. Hazard. Mater..

[8] Pan M., Chu L.M. (2016). Phytotoxicity of veterinary antibiotics to seed germination and root elongation of crops. Ecotoxicol. Environ. Saf..

[9] Guo Q., Qi H.Z., Zhou Y.P. (2012). Metabonomics and the research of traditional Chinese Medicine. Adv. Mater. Res..

[10] Carreño-Carrillo C.V., Sánchez E.V., Verduzco C.V., Herbert-Pucheta J.E. (2021). Polyphenol-based nuclear magnetic resonance non-targeted metabolomics of temperature- and time-controlled blue and red maize sprouting. SN App. Sci..

[11] Liu J.L., Liu Y.H., Lin H.Q., Zhou B.S., Yu H., Li L., Wang C.X., Li X.Y., Li P.Y., Liu J.P. (2021). The effect of ginsenoside Rg5, isolated from black ginseng, on heart failure in zebrafish based on untargeted metabolomics. J. Funct. Foods.

[12] Jin J.J. (2023). Ecotoxicological mechanism of sulfamethoxazole on Chinese cabbage and rice seedlings. Zhejiang Gongshang Univ..

[13] Ren J.W., Lu H.B., Lu S.Y., Huang Z.G. (2024). Impacts of sulfamethoxazole stress on vegetable growth and rhizosphere bacteria and the corresponding mitigation mechanism. Front. Bioeng. Biotechnol..

[14] Zhao M.T., Li J., Zhou S.S., Li K., Niu L.L., Zhao L., Xu D.M. (2023). Analysis of the effects of sulfamethoxazole on the secondary metabolites and antioxidants in oilseed rape (*Brassica napus* L.) and the underlying mechanisms. Sci. Total Environ..

[15] Feng P.F., Cui H.W., Wang C.Y., Li X.Y., Duan W.Y. (2023). Oxidative stress responses in two marine diatoms during sulfamethoxazole exposure and the toxicological evaluation using the IBRv2 index. Comp. Biochem. Phys. C.

[16] Zhang T.X., Song Y., Xu W.Y., Lu N.N., Chen Y., Jia R.B., Sun S.H. (2023). Individual and combined toxicity of sulfamethoxazole and desethylatrazine to *Chlorella vulgaris*: Growth inhibition, photosynthetic activity and oxidative stress. Chem. Ecol..

[17] Li Y.N., Chen C., Li G.D., Liu Q.Y., Tian L.L. (2013). Effects of Sulfamethoxazole (SMZ) on the Content of Chlorophylll (CHL) and Soluble Protein (SP), and the Superoxide Dismutases (SOD) Activity of Wheat, Triticum aestivum. Asian J. Ecotox..

[18] Song G., Gao Y., Wu H., Hou W., Zhang C., Ma H. (2012). Physiological effect of anatase TiO_2_ nanoparticles on Lemna minor. Environ. Toxicol. Chem..

[19] Shih C.M., Ko W.C., Wu J.S., Wei Y.H., Wang L.F., Chang E.E., Lo T.Y., Cheng H.H., Chen C.T. (2004). Mediating of caspase-independent apoptosis by cadmium through the mitochondria-ROS pathway in MRC-5 fibroblasts. J. Cell. Biochem..

[20] Serrander L., Cartier L., Bedard K., Banfi B., Lardy B., Plastre O., Sienkiewicz A., Fórró L., Schlegel W., Krause K.H. (2007). NOX4 activity is determined by mRNA levels and reveals a unique pattern of ROS generation. Biochem. J..

[21] Rasool S., Ahmad A., Siddiqi T.O., Ahmad P. (2013). Changes in growth, lipid peroxidation and some key antioxidant enzymes in chickpea genotypes under salt stress. Acta Physiol. Plant.

[22] Weisany W., Sohrabi Y., Heidari G., Siosemardeh A., Ghassemi-Golezani K. (2012). Changes in antioxidant enzymes activity and plant performance by salinity stress and zinc application in soybean (*Glycine max* L.). Plant Omics J..

[23] Liu Y.D., Tang N., Lin D.B., Deng W., Li Z.G. (2023). Integration of multi-omics analyses highlights the secondary metabolism response of tomato fruit to low temperature storage. Food Res. Int..

[24] Han T., Wang B.S., Wu Z.N., Dai C.Y., Zhao J.J., Mi Z.R., Lv Y., Zhang C., Miao X.Y., Zhou J.G. (2021). Providing a view for toxicity mechanism of tetracycline by analysis of the connections between metabolites and biologic endpoints of wheat. Ecotoxicol. Environ. Saf..

[25] Khan N., Naqvi F.N. (2010). Effect of water stress on lipid peroxidation and antioxidant enzymes in local bread ryegrass hexaploids. J. Sci. Food Agric..

[26] Zeeshan M., Iqbal A., Salam A., Hu Y.X., Khan A.H., Wang X., Miao X.R., Chen X.Y., Zhang Z.X., Zhang P.W. (2024). Zinc oxide nanoparticle-mediated root metabolic reprogramming for arsenic tolerance in Soybean. Plants.

[27] Hu X., Gao Y., Fang Z. (2016). Integrating metabolism analysis with biologic endpoints provides view into nanotoxicological mechanisms of graphene oxide: From effect onset to cessation. Carbon.

