# Supplementary figures and images for: Integrated Analysis of Metabolites and Biological Endpoints Bring New Insights into Sulfamethoxazole Stress Tolerance in Ryegrass
Source: Plants (Basel). 2025 Feb 10;14(4):538. doi: 10.3390/plants14040538 (PMC11859951; doi:10.3390/plants14040538)

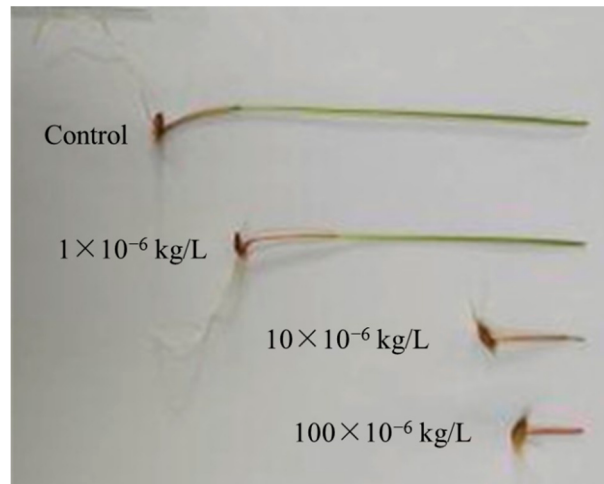

Figure S1 Seedling phenotypes.

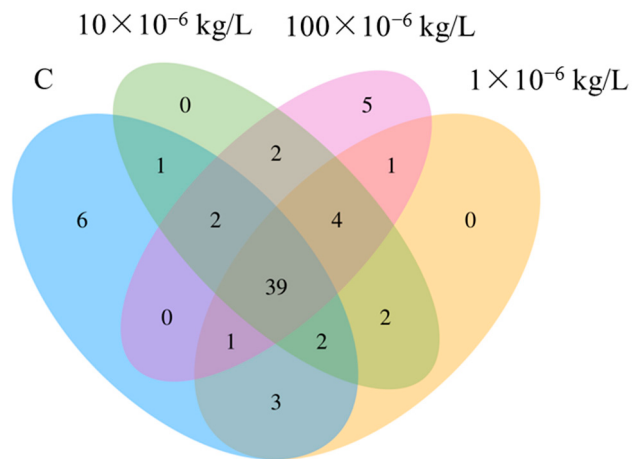

Figure S2 Venn analysis of metabolites.

Supplement: Supplementary file 1 [file plants-14-00538-s001.zip › plants-3421162-Supplementary Material.pdf]
